# Supplementary material for: Electromagnetic wave-based extreme deep learning with nonlinear time-Floquet entanglement
Source: Nat Commun. 2022 May 12;13:2651. doi: 10.1038/s41467-022-30297-5 (PMC9098897; doi:10.1038/s41467-022-30297-5)
Supplement: Supplementary file 1 — Supplementary information [file 41467_2022_30297_MOESM1_ESM.pdf]

Supplementary material for the paper:

Electromagnetic wave-based extreme deep learning with nonlinear time-Floquet entanglement

Ali Momeni, Romain Fleury

Contents

1- Time-Floquet system 2

2- Time-Floquet layer with dynamical modulation depth 4

3- Comparison of the proposed computing system with state-of-the-art works 6

4- Realistic physical platforms for the time-Floquet layer 7

1 Time-Floquet system

The transfer matrix equation relating the amplitudes of the fields on opposite sides of the time-Floquet system (See Supplementary Figure 1) can be expressed in multiplicative form in time-domain for each excitation frequency as

$$\begin{bmatrix} \mathrm{a}_1(t) \\ \mathrm{b}_1(t) \end{bmatrix} = \tilde{\Psi}(\omega_k, t) \begin{bmatrix} \mathrm{a}_2(t) \\ \mathrm{b}_2(t) \end{bmatrix} \tag{1}$$

where $\tilde{\Psi}(\omega_k, \mathrm{t})$ is the time-varying transfer matrix. The transfer matrix equation can be taken into angular frequency domain by taking the Fourier transform of both sides as:

$$\begin{bmatrix} \mathrm{A}_1(\omega) \\ \mathrm{B}_1(\omega) \end{bmatrix} = \tilde{\Psi}(\omega_k, \omega) * \begin{bmatrix} \mathrm{A}_2(\omega) \\ \mathrm{B}_2(\omega) \end{bmatrix} = \int \tilde{\Psi}(\omega_k, \omega - \omega') \begin{bmatrix} \mathrm{A}_2(\omega') \\ \mathrm{B}_2(\omega') \end{bmatrix} \mathrm{d}\omega' \tag{2}$$

Equation (2) implies that an input frequency ω_k will be converted to a spectrum of output frequencies. In the time-Floquet system, when the elements are varying in time with a periodic modulation having a modulation frequency of ω_m, $\varepsilon_\mathrm{r} = \varepsilon_\mathrm{s} + \delta_\mathrm{m} \cos(\omega_\mathrm{m} t)$, the transfer matrix is also periodic and can be expanded into a Fourier series as $\tilde{\Psi}(\mathrm{t}) = \sum_n \tilde{\Psi}^\mathrm{n}(\omega_k) \mathrm{e}^{in\omega_\mathrm{m} t}$. And the Fourier transform takes the following form:

$$\tilde{\Psi}(\omega) = \sum_n \tilde{\Psi}^n(\omega_k) \delta(\omega - n\omega_\mathrm{m}) \tag{3}$$

By choosing $\omega = \omega_q = \omega_k + q\omega_\mathrm{m}$, $\omega' = \omega_p = \omega_k + p\omega_\mathrm{m}$, q and $p \in \{..., -1, 0, +1, ...\}$, and substituting equations (3) into (2), we arrive at the following equations[1]:

$$\left\{ \begin{bmatrix} \mathrm{A}_1(\omega_q) \\ \mathrm{B}_1(\omega_q) \end{bmatrix} \right\} = \{\tilde{\Psi}^{q-p}(\omega_k)\} \left\{ \begin{bmatrix} \mathrm{A}_2(\omega_p) \\ \mathrm{B}_2(\omega_p) \end{bmatrix} \right\} \tag{4}$$

Now, let us consider adding a phase delay of ϕ to the sinusoidal modulation profile. Writing Equation (4)

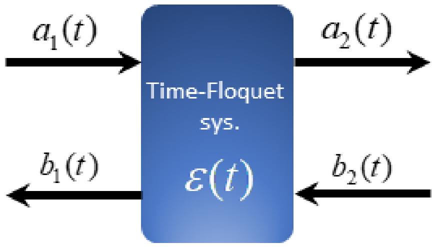

Supplementary Figure 1. The schematic of a generic two-port time-Floquet system. Incident and reflected signals at ports 1 and 2 are represented by their time-varying complex amplitudes $a_{1,2}(t)$, and $b_{1,2}(t)$.

for the phase-delayed modulation and using $\tilde{\Psi}_{\phi}^{q-p}(\omega_k) = \exp(\mathrm{i}(q-p)\phi)\tilde{\Psi}_{\phi}^{q-p}(\omega_k)$, we have:

$$\left\{ \begin{bmatrix} \mathrm{A}_1^{(\phi)}(\omega_q) \\ \mathrm{B}_1^{(\phi)}(\omega_q) \end{bmatrix} \right\} = \{\mathrm{e}^{(\mathrm{i}(q-p)\phi)}\tilde{\Psi}^{q-p}(\omega_k)\} \left\{ \begin{bmatrix} \mathrm{A}_2^{(\phi)}(\omega_p) \\ 0 \end{bmatrix} \right\} \tag{5}$$

By setting $\mathrm{B}_2(\omega_p) = 0$ for all p's and $\mathrm{A1}(\omega_q) = 0$ for all q's except $\mathrm{A}_1(\mathrm{w}_0) = 1$, we can solve the equation to obtain the reflection and transmission coefficients of all generated frequency harmonics for a monochromatic excitation of ω_k incident as:

$$\mathrm{R}_{\phi}(\omega_k + n\omega_{\mathrm{m}}) = \mathrm{e}^{in\phi}\mathrm{R}_0(\omega_k + n\omega_{\mathrm{m}}) \tag{6}$$

$$\mathrm{T}_{\phi}(\omega_k + n\omega_{\mathrm{m}}) = \mathrm{e}^{in\phi}\mathrm{T}_0(\omega_k + n\omega_{\mathrm{m}}) \tag{7}$$

where $\mathrm{R}_0(\omega_k + n\omega_{\mathrm{m}}) = \mathrm{B}_1(\omega_k + n\omega_{\mathrm{m}})/\mathrm{A}_1(\omega_k)$ and $\mathrm{T}_0(\omega_k + n\omega_{\mathrm{m}}) = \mathrm{A}_2(\omega_k + n\omega_{\mathrm{m}})/\mathrm{A}_1(\omega_k)$.

2 Time-Floquet layer with dynamical modulation depth

As an alternative approach to reach a highly nonlinear input-output mapping, we can entangle the modulation depth instead of the modulation phase with the input data (i.e., $\delta = \mathrm{f}(\zeta^{\mathrm{in}})$, where f is a linear function). In this case, the input information is encoded in the modulation depth of the Floquet layer in order to induce the required nonlinear entanglement, and no phase shifter is needed. Similar to the entanglement of modulation phase , the value of the modulation depth is directly determined by the value of the input data, which is fixed when the system is excited, automatically making the scattering process a highly non-linear function of the input, regardless of the input power. As an example, here, we show the efficiency of this approach by interpolating a highly nonlinear function ($\mathrm{sinc}(x)$) similar to section 2.1 of the main paper (see Supplementary Figure 2). In addition, similar PCA analyses on the new kernel compared with linear and square-law nonlinearity (x^2) cases are demonstrated and its corresponding results are shown in Supplementary Figure 2. As it clear from Supplementary Figure 2(e), the modulation depth based time-Floquet entanglement is extremely good at performing interpolation problems.

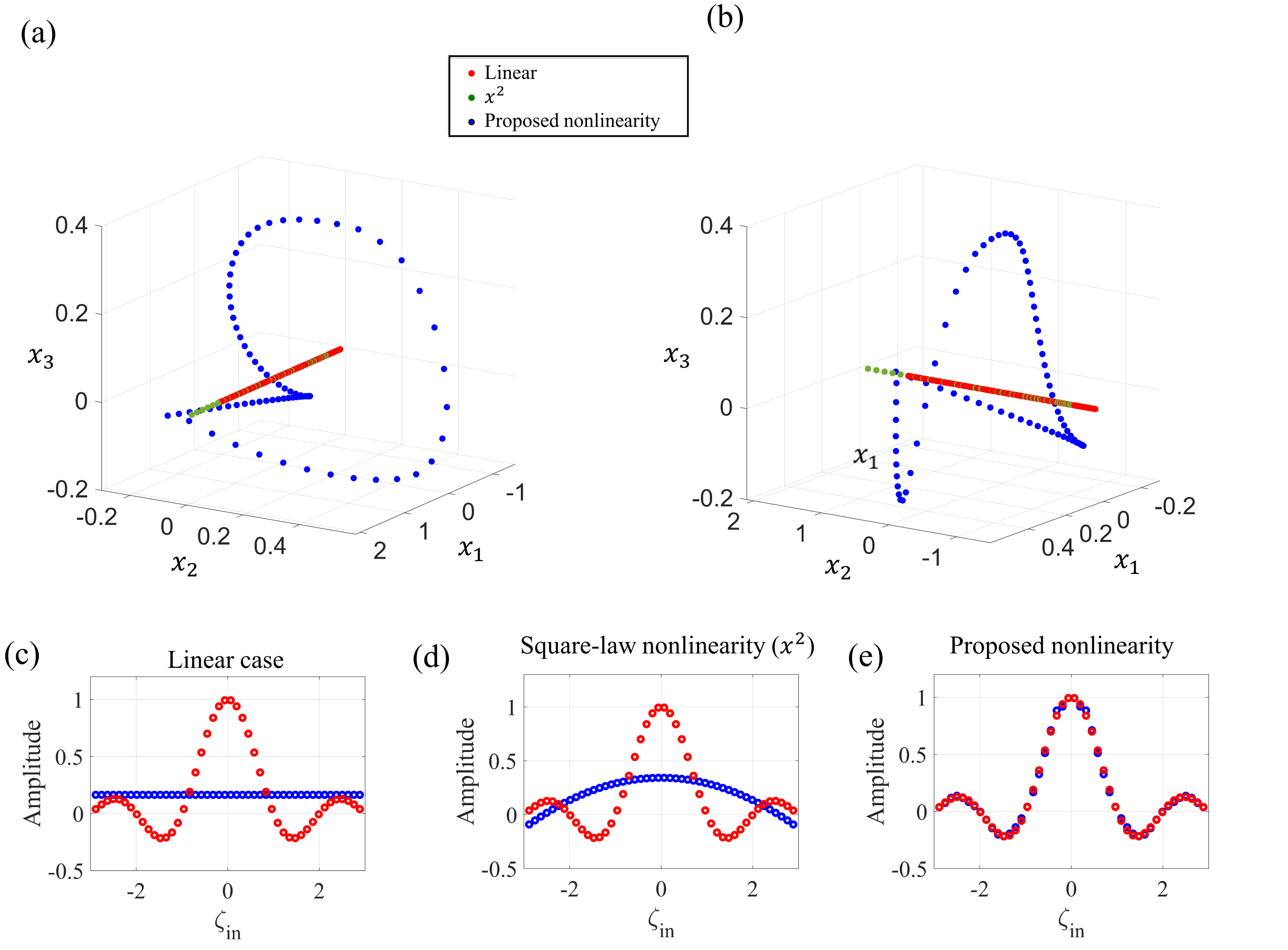

Supplementary Figure 2. PCA analysis of the proposed optical kernel for dynamical depth modulation case. (a) and (b) Two different perspectives of projected data for three different cases: i) linear case, ii) square-law nonlinearity (x^2), and iii) the proposed nonlinearity. (c), (d), and (e) sinc(ζ_{in}) interpolation results for three aforementioned cases, respectively.

3 Comparison of the proposed computing system with state-of-the-art works

Here, we compare the performance of the proposed computing system to prior works. The accuracy and RMSE comparison between the computation architectures are presented in the Supplementary Table 1.

Supplementary Table 1: Comparison of the proposed computing system with prior works

Tasks	Benchmark	Details
**Learning non-linear function (sinc(x))**	Ref [2]: RMSE: 0.0039<br>Our work: RMSE: 0.0015	• Ref [2] used a GRIN 50/125 multimode fiber (MMF) that supports 240 spatial modes.<br>• In this work, the authors used a high power optical pulse in order to excite the nonlinearity of GRIN MMF (input optical peak power pulse equal to 3.43kW).
**Abalone dataset**	Ref [2]: RMSE:0.126<br>Our work: RMSE: 0.064	• We used only 20 and 50 readout nodes for interpolating nonlinear functions and the Abalone dataset, respectively, with no power constraint.
**Parallel image classifications**	Ref [3]: Acc. (Mnist): 79.2%<br>Ref [2]: Acc. (Covid): 83.2%<br>Our work: Acc. (Mnist) 85.3%<br>Acc. (Covid): 88.2%	• The Ref [3] and [2] used all image pixels (for example 28*28 for MNIST dataset) to encode input images.<br>• We perform parallel training after down-sampling of input images (for example 10*10 for MNIST dataset). Yet, the classification-accuracy results remain comparable.
**Forecasting the chaotic Mackey Glass time series**	Ref [4]: Autonomous forecasting: for 50 time-steps<br>Our work: Autonomous forecasting: for 60 time-steps	• Ref [4] employed multiple tungsten oxide (WOx) memristors as a reservoir computing system. In this work, the authors used 20 devices (memristors) and 50 virtual nodes (total 1000 nodes) to Forecast the chaotic Mackey Glass time series.<br>• We used only 100 and 50 input and readout nodes, respectively.

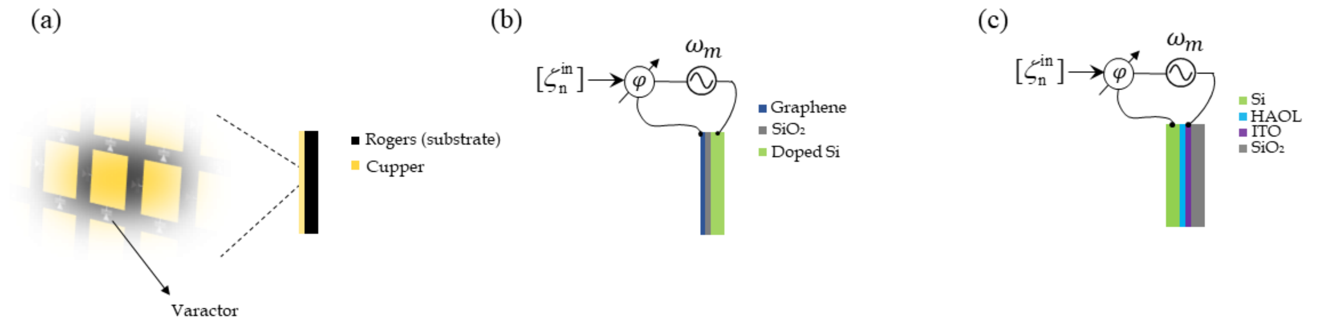

Supplementary Figure 3. Realistic physical platforms to implement the time-Floquet layer at (a) Microwave frequencies; (b), in the terahertz range; and (c), in optics.

4 Realistic physical platforms for the time-Floquet layer

In this section, we discuss realistic physical platforms for the time-Floquet layer in more details. Note that the modulation frequency is small compared to the operating frequencies f_1 and f_2, since $f_m = |f_1 - f_2|/2$, and the modulation depth does not have to be large, as long as one can detect the Floquet harmonics above the noise level. This flexibility allows the proposed time-Floquet layer to be implemented in different frequency ranges.

At ***microwave*** frequencies, one can leverage a metasurface that incorporates a single temporally modulated capacitive layer backed by a dielectric layer. As a simple example, consider periodically arranged square patches (or other form of footprint patterns) with varactors soldered between the neighboring patches (see Supplementary Figure 3(a)). The time-varying modulation $C(t) = C_0(1-\delta\sin(\omega_m t + \phi))$ is introduced by applying a time-varying voltage to the varactors[5–10].

In the ***terahertz*** and mid-infrared band, graphene is a good candidate to implement time-varying components due to its tunable electrical conductivity and compatibility with common micro-fabrication technologies. The sheet conductivity of graphene can be effectively modified via electrical bias[11,12](see Supplementary Figure 3(b)). We can model graphene as an infinitesimally thin sheet with surface impedance $Z = 1/\sigma_g$, where σ_g is the frequency-dependent complex conductivity of graphene. The surface conductivity of graphene including both intraband (σ_{intra}) and interband (σ_{inter}) transitions are governed

by the well-known Kubo formula[13]

$$\sigma_{\mathrm{g}}(\omega,\tau,\mu_{\mathrm{c}},T)=\sigma_{\mathrm{intra}}(\omega,\tau,\mu_{\mathrm{c}},T)+\sigma_{\mathrm{inter}}(\omega,\tau,\mu_{\mathrm{c}},T), \tag{8}$$

$$\sigma_{\mathrm{intra}}(\omega,\tau,\mu_{\mathrm{c}},T)=-\mathrm{j}\frac{e^2k_BT}{\pi\hbar^2(\omega-\mathrm{j}\tau^{-1})}\left(\frac{\mu_{\mathrm{C}}}{k_BT}+2\ln\left(e^{-\mu_{\mathrm{c}}/k_BT}+1\right)\right), \tag{9}$$

$$\sigma_{\mathrm{inter}}(\omega,\tau,\mu_{\mathrm{c}},\mathrm{T})=-\mathrm{j}\frac{e^2}{4\pi\hbar}\ln\left(\frac{2\mu_{\mathrm{C}}-(\omega-\mathrm{j}\tau^{-1})\hbar}{2\mu_{\mathrm{C}}+(\omega-\mathrm{j}\tau^{-1})\hbar}\right), \tag{10}$$

where e, $\hbar$, and k_B are constants corresponding to electron charge, the reduced Planck's constant, and the Boltzmann constant, respectively[13]. In the above equation, variables T, τ, and μ_{c} correspond to the environmental temperature, relaxation time, and the chemical potential of the graphene, and ω is the radian angular frequency[13]. In the proposed structure (Supplementary Figure 3(b)), the unpatterned graphene layer is a lossy medium that can be modeled through a series RL circuit in the transmission line model. The frequency-dependent resistance and inductance can be calculated by[14]

$$R(\omega,\tau,\mu_{\mathrm{c}},T)=\frac{\pi\hbar^2}{\mathrm{e}^2k_BT\left(\frac{\mu_{\mathrm{C}}}{k_BT}+2\ln\left(e^{-\mu_{\mathrm{c}}/k_BT}+1\right)\right)}, \tag{11}$$

$$L(\omega,\tau,\mu_{\mathrm{c}},T)=\frac{\pi\hbar^2}{e^2k_BT\tau\left(\frac{\mu_{\mathrm{C}}}{k_BT}+2\ln\left(e^{-\mu_{\mathrm{c}}/k_B.T}+1\right)\right)}. \tag{12}$$

If a temporally varying gate voltage is applied on the graphene sheet in Supplementary Figure 3(b), both its sheet resistance (R) and its inductance (L) will be harmonically modulated around their static values[15–18].

In the ***optical*** domain, there are two main methods to achieve the needed time-varying responses. The first method is to apply a time-varying voltage on special materials such as indium tin oxide (ITO) as

an electro-optical material and utilize Al_2O_3/HfO_2 nanolaminates (HAOL) (see Supplementary Figure 3(c)). ITO is one of the most well-known transparent conducting oxides for realization of electro-optical modulators used in telecommunications[19]. The degenerate doping of ITO (ranging from 10^{19} to 10^{21} cm^{-3}) can redshift its plasma frequency into infrared leading to a largely tunable optical response in infrared frequencies. Similar to graphene, here, by applying the time-varying voltage, dynamic phase modulation in reflection and transmission can be achieved[20,21]. Another approach leverages the changes in the phase shifts induced by the optical pump which enables fast temporal phase modulation. In this approach, two laser lines that are closely spaced in frequency results in a travelling-wave. Projecting this interference pattern on the metasurface/metamaterils imprints a travelling-wave phase profile onto the reflected wave[22].

Supplementary References

1. Salary, M. M., Jafar-Zanjani, S. & Mosallaei, H. Electrically tunable harmonics in time-modulated metasurfaces for wavefront engineering. *New J. Phys.* **20**, 123023 (2018).
2. Teğin, U., Yıldırım, M., Oğuz, İ., Moser, C. & Psaltis, D. Scalable optical learning operator. *arXiv preprint arXiv:2012.12404* (2020).
3. Nakajima, M., Tanaka, K. & Hashimoto, T. Scalable reservoir computing on coherent linear photonic processor. *Commun. Phys.* **4**, 1–12 (2021).
4. Moon, J. *et al.* Temporal data classification and forecasting using a memristor-based reservoir computing system. *Nat. Electron.* **2**, 480–487 (2019).
5. Zhang, L. *et al.* Space-time-coding digital metasurfaces. *Nat. communications* **9**, 1–11 (2018).
6. Zhang, L. *et al.* Breaking reciprocity with space-time-coding digital metasurfaces. *Adv. Mater.* **31**, 1904069 (2019).
7. Wang, X. *et al.* Nonreciprocity in bianisotropic systems with uniform time modulation. *Phys. Rev. Lett.* **125**, 266102 (2020).
8. Sounas, D. L. & Alu, A. Non-reciprocal photonics based on time modulation. *Nat. Photonics* **11**, 774–783 (2017).
9. Taravati, S. & Eleftheriades, G. V. Microwave space-time-modulated metasurfaces. *ACS Photonics* (2022).
10. Taravati, S. & Eleftheriades, G. V. Full-duplex nonreciprocal beam steering by time-modulated phase-gradient metasurfaces. *Phys. Rev. Appl.* **14**, 014027 (2020).
11. Ju, L. *et al.* Graphene plasmonics for tunable terahertz metamaterials. *Nat. nanotechnology* **6**, 630–634 (2011).
12. Phare, C. T., Lee, Y.-H. D., Cardenas, J. & Lipson, M. Graphene electro-optic modulator with 30 ghz bandwidth. *Nat. Photonics* **9**, 511–514 (2015).

13. Hanson, G. W. Dyadic green's functions and guided surface waves for a surface conductivity model of graphene. *J. Appl. Phys.* **103**, 064302 (2008).

14. Momeni, A., Rouhi, K. & Fleury, R. Switchable and simultaneous spatiotemporal analog computing with computational graphene-based multilayers. *Carbon* **186**, 599–611 (2022).

15. Wang, X., Díaz-Rubio, A., Li, H., Tretyakov, S. A. & Alù, A. Theory and design of multifunctional space-time metasurfaces. *Phys. Rev. Appl.* **13**, 044040 (2020).

16. Rajabalipanah, H., Fakheri, M. H. & Abdolali, A. Electromechanically programmable space-time-coding digital acoustic metasurfaces. *arXiv preprint arXiv:2003.12616* (2020).

17. Sedeh, H. B., Salary, M. M. & Mosallaei, H. Active multiple access secure communication enabled by graphene-based time-modulated metasurfaces. *IEEE Transactions on Antennas Propag.* **70**, 664–679 (2021).

18. Salary, M. M., Jafar-Zanjani, S. & Mosallaei, H. Time-varying metamaterials based on graphene-wrapped microwires: Modeling and potential applications. *Phys. Rev. B* **97**, 115421 (2018).

19. Wuttig, M., Bhaskaran, H. & Taubner, T. Phase-change materials for non-volatile photonic applications. *Nat. Photonics* **11**, 465–476 (2017).

20. Barati Sedeh, H., Salary, M. M. & Mosallaei, H. Topological space-time photonic transitions in angular-momentum-biased metasurfaces. *Adv. Opt. Mater.* **8**, 2000075 (2020).

21. Salary, M. M., Farazi, S. & Mosallaei, H. A dynamically modulated all-dielectric metasurface doublet for directional harmonic generation and manipulation in transmission. *Adv. Opt. Mater.* **7**, 1900843 (2019).

22. Guo, X., Ding, Y., Duan, Y. & Ni, X. Nonreciprocal metasurface with space–time phase modulation. *Light. Sci. & Appl.* **8**, 1–9 (2019).
